# Prevalence of use, perceptions of harm and addictiveness, and dependence of electronic cigarettes among adults in Kuwait: A cross-sectional study

**DOI:** 10.18332/tid/163300

**Published:** 2023-07-14

**Authors:** Munairah Alshaibani, Mays Alajmi, Noura Alabduljalil, Hajar Alajmi, Yousef Alsalem, Danah Aloqab, Hamad Alawadhi, Sara Sayed Ali, Yaqoub Sharhan, Ahmed Alzeeny, Ali H. Ziyab

**Affiliations:** 1 Department of Community Medicine and Behavioral Sciences, Faculty of Medicine, Kuwait University, Kuwait City, Kuwait

**Keywords:** electronic cigarettes, adults, dependence, harm, addictiveness

## Abstract

**INTRODUCTION:**

Electronic cigarettes (e-cigarettes) have become one of the most common forms of nicotine delivery used by youth and young adults worldwide. Given the lack of epidemiologic data in Kuwait, this study sought to estimate the prevalence of e-cigarette use, assess perceptions of harm and addictiveness of e-cigarettes, measure the level of dependence on e-cigarettes and assess factors associated with dependence level among adults.

**METHODS:**

A cross-sectional study enrolled adults (n=3032, aged ≥18 years) living in Kuwait using a web-based questionnaire. Participants self-reported ever and current (past 30-day) e-cigarette use and self-completed the 10-item Penn State E-cigarette Dependence Index. Associations were evaluated using multinomial logistic regression.

**RESULTS:**

The prevalence of ever and current e-cigarette use was estimated to be 40.2% (1220/3032) and 29.4% (892/3032), respectively. The prevalence of current e-cigarette use was higher in males compared to females (47.6% vs 14.4%, p<0.001). Relative to cigarette smoking, 40.6% of participants reported that e-cigarettes are less harmful, and 41.8% indicated that e-cigarettes are equally addictive. Among current e-cigarette users (n=892), 84.8% were ascertained to have developed either low, medium, or high dependence. The use of pod-based devices compared to disposable devices was associated with a high dependence level (adjusted odds ratio, AOR=8.56; 95% CI: 4.52–16.22).

**CONCLUSIONS:**

These findings suggest that a large proportion of adults in Kuwait use e-cigarettes, and a considerable proportion of current users have developed dependence. Therefore, such results should alert public health authorities and warrant the development of evidence-based awareness campaigns, policies, and prevention measures to protect and improve the health of people.

## INTRODUCTION

Smoking is one of the leading public health threats around the world as well as being a critical preventable risk factor that accounted for 8.71 million deaths and 229.77 million disability-adjusted life years (DALYs) globally in 2019^1^. Smoking has been causally associated with a wide range of health problems, such as malignancies, particularly upper airway and lung cancers, cardiovascular and metabolic diseases, pulmonary diseases, and various congenital anomalies^2^. Worldwide in 2015, the prevalence of daily smoking was estimated to be 25.0% among men and 5.4% among women^3^.

Although decreasing global trends in the prevalence of cigarette smoking have been observed over the past decades^3^, an increase in the use of electronic cigarettes (e-cigarettes), also referred to as electronic nicotine delivery systems, vaporizers, hookah pens, vape pens, and electronic pipes, has been witnessed globally among youth and adults^4,5^. In contrast to cigarettes, e-cigarettes lack the combustion of tobacco and work by evaporating a liquid, which is rapidly condensed into an aerosol (vapor) and is then inhaled by the user^6^. Flavors and nicotine are often added to the liquids used in e-cigarettes, which increase the desire to use and the dependence on these products^6^. Aerosols (vapor) emitted by e-cigarettes contain ultrafine particles, such as volatile carbonyls, formaldehyde, acetaldehyde, acrolein, and acetone, all of which have established negative health effects^6^. The use of e-cigarettes has been reported to be associated with respiratory diseases, such as asthma^7^ and chronic obstructive pulmonary disease symptoms^8^; nevertheless, causal evidence is inconclusive. Compared to cigarette smoking, less is known about the epidemiology and the short- and long-term health effects of e-cigarettes.

Although Kuwait ratified the World Health Organization Framework Convention on Tobacco Control (WHO FCTC) in 2006 and issued and implemented several laws to control tobacco use (e.g. banning smoking in public places, workplaces, and public transportation, prohibiting marketing and promotion of tobacco products, requiring warning labels on tobacco product packages), the burden of tobacco smoking remains substantial in Kuwait. A study based on a nationally representative sample estimated the prevalence of current (past 30-day) cigarette smoking to be 39.2% among men and 3.3% among women^9^. Likewise, among adolescents in Kuwait, it has been estimated that 25.1% (males: 45.1%; females: 8.4%) are current cigarette smokers^10^. With regard to e-cigarette use, an estimated prevalence of current use was reported to be 26.4% among adolescents in Kuwait, with more males than females being current e-cigarette users (46.8% vs 9.3%)^10^. Currently, there are no regulations on e-cigarettes in Kuwait, except the minimum age for purchasing, which is 21 years. There is a need for regulations that ban the use of e-cigarettes in workplaces, public places, and on public transportation, and prohibit media promotions and advertising of e-cigarettes. In Kuwait, data on the epidemiology of e-cigarettes among adults are lacking. Hence, to better inform public health policies, regulations, and campaigns, this study sought to: 1) estimate the prevalence of e-cigarette use among adults in Kuwait, 2) examine how adults in Kuwait perceive the harm and addictiveness of e-cigarettes in general and relative to conventional cigarettes; and 3) assess the level of dependence among current users of e-cigarettes and determine factors associated with dependence levels.

## METHODS

### Study setting, design, and participants

A cross-sectional study was conducted whereby adults (n=3032, aged ≥18 years) living in Kuwait were recruited through social media platforms (WhatsApp, Instagram, Snapchat, and Twitter) from 27 January to 2 February 2021. Invitations to participate were disseminated on these platforms, and participants were asked to further disseminate the survey to their acquaintances. Hence, the snowball sampling technique, a non-probability sampling method that yields a convenience sample, was used to recruit participants. The invitation text indicated that only individuals living in Kuwait and aged ≥18 years should participate. The study was approved by the Health Sciences Center Ethics Committee for Student Research at Kuwait University. Completing the questionnaire was deemed informed consent to participate. The study was conducted in accordance with the principles and guidelines of the Declaration of Helsinki for medical research involving human subjects.

### Study questionnaire and variable definitions

The study questionnaire, designed to be self-completed, collected information on sociodemographic factors, perceptions about the harmfulness and addictiveness of e-cigarettes, use of e-cigarettes, cigarette smoking, and measured dependence on e-cigarettes. The sociodemographic characteristics that were measured included age group, sex, education level, marital status, employment status, nationality, governorate/region of residence, and family’s total monthly income in Kuwaiti Dinar.

The study questionnaire was originally developed using the English language, which was translated into the Arabic language by two of the study investigators who are Arabic native speakers and proficient in the English language. The Arabic-translated version of the study questionnaire was checked by the other investigators to ensure consistency with the original version. Subsequently, pilot testing of the Arabic version of the study questionnaire was conducted. The respondents in the pilot testing were asked to explain their understanding of the questions to confirm content validity. The comprehensibility and coherence were also reviewed, and the required modifications were done before finalizing the questionnaire. Participants completed either the English or Arabic version of the study questionnaire, as per their preference.

The perception of harm of e-cigarettes and cigarettes was assessed by asking separate questions using the following stem: ‘How much do you think people harm themselves when they smoke cigarettes/use e-cigarettes?’, with response options of no harm, little, some, or much harm. An additional response option of ‘I have never heard of e-cigarettes’ was provided for the question asking about the harm of e-cigarettes. The relative harm perception of e-cigarettes compared to cigarette smoking was assessed with the question: ‘Compared to smoking cigarettes, do you think that e-cigarettes and other vaping devices are more harmful, less harmful, or equally harmful to health?’. With a response option of less harmful, equally harmful, more harmful, I have never heard of e-cigarettes, or I do not know. The relative addiction perception of e-cigarettes compared to cigarettes was assessed with the item: ‘Do you believe that e-cigarettes are (less addictive, equally addictive, or more addictive) than cigarettes?’. With response options of less addictive, equally addictive, or more addictive, ‘I have never heard of e-cigarettes’, or ‘I do not know’. These questions were adapted from a prior study^11^, the National Youth Tobacco Survey (NYTS)^12^, and the Global Adult Tobacco Survey (GATS)^13^.

Questions from the NYTS and GATS questionnaires^12,13^ were adapted to assess the use of e-cigarettes and smoking cigarettes and related practices. Participants were asked to report if they have ever used e-cigarettes and the frequency of use, if any, in the past 30 days in terms of the number of days. Ever e-cigarette use was defined as any reported lifetime use of e-cigarettes. Current e-cigarette use was defined as any reported use of e-cigarettes in the past 30 days. Former use of e-cigarette was defined as ever use but not current use. Ever, former, and current cigarette smoking were similarly ascertained. Moreover, considering e-cigarette use and cigarette smoking concurrently, we reported never users of both products, former users of both products, current e-cigarette users only, current cigarette smokers only, and current dual users of e-cigarettes and cigarettes.

Current users of e-cigarettes were further classified as non-daily users and daily users based on their reported frequency of use in the past 30 days. Also, the number of years of using e-cigarettes was reported as <1 year versus ≥1 year. The use of nicotine in e-cigarettes was also reported. Information regarding the type of e-cigarette used in the past 30 days, including disposable, pre-filled pods or cartilages (e.g. JUUL, Phix), liquid refillable tanks, or mod systems, was also obtained. The number of times, ranging from never to more than 6 times, the participant stopped using e-cigarettes in the past 12 months was also reported. Moreover, to better understand the motives behind using e-cigarettes, participants were asked to choose the applicable reason(s) from a predefined list that included: being used by friend(s)/family member(s), trying to quit, less cost as well as easier to get than cigarettes, or seeing people on TV, online or in movies use them. Other reasons are: being regarded as less harmful, available in flavors, curiosity, used unnoticed, or to do tricks. In addition, the used flavors of e-cigarettes in the past 30 days were reported by choosing from a list.

### Ascertainment of dependence on e-cigarettes

The 10-item Penn State E-cigarette Dependence Index was used to measure the dependence levels of current e-cigarette users in our study^14^. The dependence score ranges between 0 (no dependence) and 20 (high dependence). According to Foulds et al.^14^ scoring scheme, the following groupings were considered: not dependent (score: 0–3), low dependence (score: 4–8), medium dependence (score: 9–12), and high dependence (score: ≥13). A previous study reported that the 10-item Penn State E-cigarette Dependence Index has an acceptable internal consistency (Cronbach’s α=0.74)^15^. In our study, the internal consistency of the scale was estimated to be: Cronbach’s α=0.76.

### Statistical analysis

Analyses were conducted using SAS 9.4 (SAS Institute, Cary, NC, USA). The statistical significance level was set to α=0.05 for all association analyses. Descriptive analyses were conducted to calculate frequencies and proportions of categorical variables. The dependence on e-cigarettes score, a non-normally distributed quantitative variable, was described by calculating the median and interquartile range (IQR). Chi-squared (χ^2^) test was used to assess whether the prevalence of ever and current e-cigarette use differed across categories of sociodemographic and lifestyle factors. Moreover, non-parametric tests were used to determine whether the median of e-cigarette dependence score differed across groups of categorical variables. Namely, the Wilcoxon rank sum test was used to compare the medians of two groups, and the Kruskal-Wallis test was used to determine whether the medians of three or more groups differ. Associations between different factors/characteristics (independent variables) and level of e-cigarettes dependence (ordinal outcome variable; the ‘no dependence’ group was set as the reference category) were assessed by applying a multinomial logistic regression model to estimate adjusted odds ratios (AOR) and their 95% confidence interval (CI). A separate regression model was used to assess the association between the respective e-cigarette user characteristics (exposure variable) and dependence level (outcome variable) while adjusting for sex, nationality, and governorate of residence.

## RESULTS

A total of 3032 adults living in Kuwait have participated in the current study, of whom 1373 (45.3%) were males, and 1659 (54.7%) were females (Table 1). Most participants were aged 18–24 years (52.8%). Moreover, most study participants reported attaining a Bachelor’s degree or higher (59.5%), and the majority were single (64.6%). Of the total study sample, 25.5% were former cigarette smokers, and 19.1% were current cigarette smokers (Table 1). Overall, the prevalence of ever e-cigarette use was estimated to be 40.2% (n=1220), with 10.8% (n=328) being former users and 29.4% (n=892) being current users of e-cigarettes (Table 1). The prevalence of dual use of e-cigarettes and cigarettes was estimated to be 14.0% (n=425). Of the total study participants, 15.4% (n=467) reported only using e-cigarettes, and 5.1% (n=153) reported only smoking cigarettes.

**Table 1:** Characteristics of the total study sample and prevalence of ever and current (any use in the past 30 days) use of electronic cigarettes (e-cigarettes) according to the participants characteristics, Kuwait, 2021 (N=3032)

	*Total sample*	*Ever e-cigarette use*		*Current e-cigarette use*	
	*% (n)*	*% (n)*	*p*	*% (n)*	*p*
**Overall**	100.0 (3032)	40.2 (1220)	-	29.4 (892)	-
**Sex**					
Male	45.3 (1373)	63.0 (865)	<0.001	47.6 (654)	<0.001
Female	54.7 (1659)	21.4 (355)		14.4 (238)	
**Age** (years)					
18–24	52.8 (1600)	35.4 (567)	<0.001	25.5 (408)	<0.001
25–34	25.8 (781)	54.0 (422)		40.6 (317)	
35–44	12.6 (383)	44.1 (169)		32.4 (124)	
45–54	6.4 (194)	26.3 (51)		18.0 (35)	
≥55	2.4 (74)	14.9 (11)		10.8 (8)	
**Education level**					
High school or lower	25.3 (768)	32.7 (251)	<0.001	24.4 (187)	<0.001
Diploma*	15.2 (461)	52.1 (240)		39.5 (182)	
Bachelor’s degree or higher	59.5 (1803)	40.4 (729)		29.0 (523)	
**Marital status**					
Single	64.6 (1958)	39.5 (773)	0.407	28.6 (560)	0.396
Married	31.5 (955)	41.3 (394)		30.8 (294)	
Divorced/widowed	3.9 (119)	44.5 (53)		31.9 (38)	
**Employment status**					
Student	47.3 (1435)	33.8 (485)	<0.001	24.9 (357)	<0.001
Employed	39.2 (1188)	49.0 (582)		36.3 (431)	
Unemployed	5.4 (165)	45.6 (75)		30.9 (51)	
Retired	3.6 (109)	15.6 (17)		8.3 (9)	
Private business owner	2.4 (71)	63.4 (45)		46.5 (33)	
Other	2.1 (64)	25.0 (16)		17.2 (11)	
**Nationality**					
Kuwaiti	81.3 (2465)	41.1 (1014)	0.035	30.1 (742)	0.086
Non-Kuwaiti	18.7 (567)	36.3 (206)		26.5 (150)	
**Governorate** ^†^					
Al Asimah	25.7 (731)	39.8 (291)	<0.001	28.9 (211)	<0.001
Hawalli	28.0 (798)	45.1 (260)		33.1 (264)	
Al Ahmadi	13.8 (392)	33.7 (132)		21.9 (86)	
Jahra	6.9 (197)	31.5 (62)		25.4 (50)	
Farwaniya	11.4 (324)	37.0 (120)		25.6 (83)	
Mubarak Al-Kabeer	14.2 (404)	46.8 (189)		37.9 (153)	
**Monthly household income^‡^** (KWD)					
<1000	18.6 (452)	42.0 (190)	0.724	29.7 (134)	0.242
1000–1999	39.3 (956)	42.4 (405)		32.9 (314)	
2000–2999	18.6 (453)	41.7 (189)		29.1 (132)	
≥3000	23.5 (570)	39.5 (225)		28.4 (162)	
**Cigarette smoking status**					
Never	55.4 (1681)	13.3 (223)	<0.001	8.8 (148)	<0.001
Former	25.5 (773)	59.4 (459)		41.3 (319)	
Current	19.1 (578)	93.1 (538)		73.5 (425)	

KWD: 100 Kuwaiti Dinar about 325 US$.

* Refers to a two-year associate degree post high school.

† Missing values: n=186.

‡ Missing values: n=601.

The prevalence of current e-cigarette use was higher in males than females (47.6% vs 14.4%, p<0.001) (Table 1). Moreover, participants aged 25–34 years reported the highest current use of e-cigarettes (40.6%) compared to other age groups. The prevalence of current e-cigarette use differed according to education level, employment status, and governorate of residence. Current cigarette smokers were significantly more likely than never smokers to be current e-cigarette users (73.5% vs 8.8%, p<0.001) (Table 1).

Table 2 presents data on participants’ perceptions of the harm and addictiveness of e-cigarettes and cigarettes. In total, 76.3% of participants reported that smoking cigarettes is associated with ‘much harm’, whereas 50.1% of participants reported that the use of e-cigarettes is associated with ‘much harm’. More females, compared to males, reported that cigarettes (83.8% vs 67.1%) and e-cigarettes (61.7% vs 36.1%) are associated with ‘much harm’. Moreover, participants who never used cigarettes and e-cigarettes compared to current dual users of e-cigarettes and cigarettes were more likely to associate the use of cigarettes (90.0% vs 48.0%) and e-cigarettes (67.8% vs 19.6%) with ‘much harm’. Compared to cigarettes, 40.6% of our study sample believed that e-cigarettes are ‘less harmful’ and 41.8% perceived that e-cigarettes are ‘equally addictive’ (Table 2). More males than females reported that e-cigarettes relative to cigarettes, are ‘less harmful’ (54.7% vs 29.0%) and ‘less addictive’ (34.6% vs 23.3%). Dual users, compared to never users were more likely to perceive e-cigarettes relative to cigarettes to be ‘less harmful’ (61.2% vs 26.2%) and ‘less addictive’ (40.0% vs 18.7%) (Table 2).

**Table 2:** Perceived harm of cigarette smoking and electronic cigarette (e-cigarette) use, perceived relative harm of e-cigarette use compared to cigarette smoking, and perceived relative addictiveness of e-cigarette use compared to cigarette smoking in the total study sample and according to sex and e-cigarette use and cigarette smoking, Kuwait, 2021 (N=3032)

*Perceptions*		*Sex, % (n)*		*Current e-cigarette use and cigarette smoking status, % (n)*				
	*Total sample (N=3032) % (n)*	*Male (N=1373)*	*Female (N=1659)*	*Never users (N=1458)*	*Former users (N=529)*	*E-cigarette only users (N=467)*	*Cigarette only smokers (N=153)*	*Dual users (N=425)*
**Harm of cigarettes**								
No harm	0.7 (22)	1.2 (16)	0.4 (6)	0.5 (8)	0.4 (2)	0.9 (4)	1.3 (2)	1.4 (6)
Little	3.6 (108)	5.5 (76)	1.9 (32)	0.6 (9)	0.7 (4)	7.7 (36)	6.6 (10)	11.5 (49)
Some	19.4 (589)	26.2 (359)	13.9 (230)	8.9 (129)	17.6 (93)	30.2 (141)	39.2 (60)	39.1 (166)
Much	76.3 (2313)	67.1 (922)	83.8 (1391)	90.0 (1312)	81.3 (430)	61.2 (286)	52.9 (81)	48.0 (204)
Harm of e-cigarettes*								
No harm	1.8 (54)	3.1 (42)	0.7 (12)	0.30 (5)	0.8 (4)	4.5 (21)	2.0 (3)	5.0 (21)
Little	14.2 (430)	23.1 (316)	6.9 (114)	5.1 (74)	6.8 (36)	35.3 (165)	11.1 (17)	32.6 (138)
Some	33.9 (1026)	37.7 (517)	30.7 (509)	26.8 (389)	38.0 (201)	42.4 (198)	37.2 (57)	42.8 (181)
Much	50.1 (1515)	36.1 (494)	61.7 (1021)	67.8 (985)	54.4 (288)	17.8 (83)	49.7 (76)	19.6 (83)
**Harm of e-cigarettes relative to cigarettes^†^**								
Less harmful	40.6 (1226)	54.7 (748)	29.0 (477)	26.2 (380)	37.3 (196)	74.7 (348)	27.6 (42)	61.2 (260)
Equally harmful	29.7 (895)	21.0 (288)	36.9 (607)	38.8 (562)	28.2 (148)	15.5 (72)	21.7 (33)	18.8 (80)
More harmful	20.5 (618)	16.2 (222)	24.0 (396)	24.3 (351)	22.7 (119)	6.4 (30)	40.8 (62)	13.2 (56)
Do not know	9.2 (277)	8.1 (110)	10.1 (167)	10.7 (155)	11.8 (62)	3.4 (16)	9.9 (15)	6.8 (29)
**Addictiveness of e-cigarettes relative to cigarettes^‡^**								
Less addictive	28.4 (859)	34.6 (475)	23.3 (384)	18.7 (271)	27.1 (143)	48.8 (228)	30.9 (47)	40.0 (170)
Equally addictive	41.8 (1264)	35.9 (493)	46.7 (771)	46.8 (678)	41.9 (221)	34.9 (163)	35.5 (54)	34.8 (148)
More addictive	19.8 (597)	21.6 (296)	18.2 (301)	20.1 (292)	21.2 (112)	13.5 (63)	26.3 (40)	21.2 (90)
Do not know	10.0 (302)	7.9 (108)	11.8 (194)	14.4 (209)	9.8 (52)	2.8 (13)	7.3 (11)	4.0 (17)

* Missing values: n=7.

† Missing value: n=16.

‡ Missing values: n=10.

Among current users of e-cigarettes (n=892), smoking cessation was the most frequently reported reason for the initiation of e-cigarette use (56.2%), followed by their availability in a variety of flavors (37.2%; data not shown). Moreover, nearly three-quarters (75.6%) of current e-cigarette users reported using a fruit-flavored e-cigarette (75.6%), followed by tobacco (26.6%) and mint (21.7%) flavors (data not shown). In terms of the most commonly used type of e-cigarette devices in the past 30 days, the majority of the current e-cigarette users reported the use of disposable devices (37.5%), followed by pod-based devices (28.5%), refillable devices (26.0%), and some other types (8.0%).

According to the Penn State E-cigarette Dependence Index classification, of the total current e-cigarette users (n=892), 41.3% (n=368) were classified to have low dependence, 26.9% (n=240) to have medium dependence, 16.6% (n=148) have high dependence, and 15.2% (n=136) were determined to have no dependence. Online Supplementary file Table S1 shows the frequencies of responses to the ten items used to assess dependence on e-cigarettes. Bivariate associations between the e-cigarette dependence score and sociodemographic variables are shown in Table 3. Overall, the median (IQR) e-cigarette dependence score was estimated to be 8.0 (5.0–11.0) among current users (n=892). The median e-cigarettes dependence score was higher among males compared to females (8.0 vs 6.0, p<0.001), higher among Kuwaiti than non-Kuwaiti participants (8.0 vs 6.0, p=0.002), and highest among residents of Al-Asimah governorate (Table 3).

**Table 3:** Electronic cigarettes dependence score according to personal characteristics among current electronic cigarette users, Kuwait, 2021 (N=892)

	*n*	*Dependence score Median (IQR)*	*p*
**Overall**	892	8 (5–11)	-
**Sex**			
Male	654	8 (5–12)	<0.001
Female	238	6 (4–10)	
**Age** (years)			
18–24	408	7.5 (5–12)	0.428
25–34	317	8 (5–11)	
35–44	124	7 (5–11)	
45–54	35	7 (5– 9)	
≥55	8	9.5 (5–10)	
**Education level**			
High school or lower	187	7 (5–11)	0.898
Diploma*	182	8 (5–10)	
Bachelor’s degree or higher	523	8 (5–12)	
**Marital status**			
Single	560	8 (5–12)	0.444
Married	294	7 (5–11)	
Divorced/widowed	38	7.5 (5–10)	
**Employment status**			
Student	357	8 (5–12)	0.261
Employed	431	8 (5–11)	
Unemployed	51	9 (6–11)	
Retired	9	8 (7– 8)	
Private business owner	33	6 (5– 9)	
Other	11	6 (0–10)	
**Nationality**			
Kuwaiti	742	8 (5–11)	0.002
Non-Kuwaiti	150	6 (4–10)	
**Governorate^†^**			
Al Asimah	211	9 (5–13)	0.032
Hawalli	264	7 (5–11)	
Al Ahmadi	86	7.5 (5–10)	
Jahra	50	8 (5–12)	
Farwaniya	83	7 (4–10)	
Mubarak Al-Kabeer	153	7 (5–10)	
**Monthly household Income^‡^** (KWD)			
<1000	134	7.5 (5–11)	0.908
1000–1999	314	8 (5–11)	
2000–2999	132	7 (5–12)	
≥3000	162	8 (5–11)	

KWD: 100 Kuwaiti Dinar about 325 US$. IQR: interquartile range.

* Refers to a two-year associate degree post high school.

† Missing values: n=45.

‡ Missing values: n=150.

Adjusted associations between different factors with e-cigarette dependence levels are shown in Table 4. Using e-cigarettes for ≥1 year compared to <1 year was associated with medium dependence (AOR=3.58; 95% CI: 2.39–5.37) and high dependence (AOR=5.12; 95% CI: 3.17–8.29) levels. Moreover, daily use compared to non-daily use of e-cigarettes was associated with low dependence (AOR=3.56; 95% CI: 2.13–5.95), medium dependence (AOR=10.03; 95% CI: 5.79–17.35), and high dependence (AOR=26.87; 95% CI: 13.80–52.29) levels. The use of e-cigarettes that contain nicotine was associated with low, medium, and high dependence levels. Compared to using disposable e-cigarettes, using pod-based e-cigarettes was associated with low dependence (AOR=1.90; 95% CI: 1.12–3.26), medium dependence (AOR=3.09; 95% CI: 1.76–5.44), and high dependence levels (AOR=8.56; 95% CI: 4.52–16.22) (Table 4). The use of refillable e-cigarette devices compared to disposable e-cigarette devices was also associated with dependence levels. The number of attempts to stop using e-cigarettes was found to be related to dependence levels. For example, compared to subjects who tried to stop e-cigarette use ≥6 times in the past 12 months, those who never tried to stop using e-cigarettes were more likely to have developed medium dependence (AOR=4.93; 95% CI: 2.93–8.29) and high dependence levels (AOR=4.75; 95% CI: 2.54–8.90). Similarly, compared to never-cigarette smokers, current cigarette smokers were more likely to have developed medium dependence (AOR=3.31; 95% CI: 1.92–5.70) and high dependence on e-cigarettes (AOR=4.12; 95% CI: 2.09–8.13) (Table 4).

**Table 4:** Factors associated with electronic cigarette (e-cigarette) dependence levels among current users, Kuwait, 2021 (N=892)

*Factors*		*Dependence score*		*Level of e-cigarette dependence, % (n)*			
	*n*	*Median (IQR)*	*p*	*None*	*Low*	*Medium*	*High*
**Years using e-cigarettes**							
<1	388	6 (4–9)	<0.001	21.6 (84)	49.5 (192)	18.3 (71)	10.6 (41)
≥1	495	9 (6–12)		9.9 (49)	34.6 (171)	34.1 (169)	21.4 (106)
AOR* (95% CI) [≥1 vs <1 year]				1.00 (Ref.)	1.31 (0.91–1.89)	3.58 (2.39–5.37)	5.12 (3.17–8.29)
**Frequency of use in past 30 days**							
Non-daily	404	5 (3–8)	<0.001	27.0 (109)	50.2 (203)	17.6 (71)	5.2 (21)
Daily	488	10 (7–13)		5.6 (27)	33.8 (165)	34.6 (169)	26.0 (127)
AOR* (95% CI) [daily vs non-daily]				1.00 (Ref.)	3.56 (2.13–5.95)	10.03 (5.79–17.35)	26.87 (13.80–52.29)
**E-cigarette contains nicotine**							
No	28	4 (0.5–9)	<0.001	50.0 (14)	21.4 (6)	17.9 (5)	10.7 (3)
Yes	752	8 (5–12)		11.6 (87)	41.3 (311)	28.9 (217)	18.2 (137)
AOR* (95% CI) [yes vs no]				1.00 (Ref.)	3.01 (1.39–6.53)	4.01 (1.60–10.04)	6.83 (1.88–24.85)
**Type of e-cigarette used in past 30 days**							
Disposable	332	6 (4–10)	<0.001	22.0 (73)	46.1 (153)	22.9 (76)	9.0 (30)
Refillable	230	8 (5–11)		13.9 (32)	40.4 (93)	30.9 (71)	14.8 (34)
Pod-based	252	10 (7–13)		7.1 (18)	32.5 (82)	30.2 (76)	30.2 (76)
Other or unknown	71	6 (5–10)		14.1 (10)	53.5 (38)	22.5 (16)	9.9 (7)
AOR* (95% CI) [refillable vs disposable]				1.00 (Ref.)	1.65 (1.00–2.73)	2.17 (1.25–3.74)	3.01 (1.53–5.91)
AOR* (95% CI) [pod-based vs disposable]				1.00 (Ref.)	1.90 (1.12–3.26)	3.09 (1.76–5.44)	8.56 (4.52–16.22)
AOR* (95% CI) [other vs disposable]				1.00 (Ref.)	2.19 (1.11–4.35)	1.59 (0.72–3.50)	1.55 (0.53–4.52)
**Stopped e-cigarette use in past 12 months** (times)							
≥6	166	5 (3–7)	<0.001	28.9 (48)	52.4 (87)	10.9 (18)	7.8 (13)
3–5	129	8 (5–11)		14.7 (19)	41.1 (53)	31.8 (41)	12.4 (16)
1–2	214	8.5 (6–12)		10.8 (23)	39.2 (84)	28.5 (61)	21.5 (46)
Never	383	9 (5–12)		12.0 (46)	37.6 (144)	31.3 (120)	19.1 (73)
AOR* (95% CI) [3–5 vs ≥6]				1.00 (Ref.)	1.55 (0.8–2.73)	5.86 (2.98–11.51)	4.09 (1.79–9.35)
AOR* (95% CI) [1–2 vs ≥6]				1.00 (Ref.)	1.67 (1.06–2.62)	5.07 (2.84–9.05)	5.76 (2.92–11.37)
AOR* (95% CI) [never vs ≥6]				1.00 (Ref.)	1.40 (0.95–2.07)	4.93 (2.94–8.29)	4.75 (2.54–8.90)
**Cigarette smoking status**							
Never	148	6 (3.5–9)	<0.001	25.0 (37)	48.6 (72)	16.9 (25)	9.5 (14)
Former	319	8 (5–11)		13.8 (44)	39.8 (127)	28.2 (90)	18.2 (58)
Current	425	8 (5–12)		12.9 (55)	39.8 (169)	29.4 (125)	17.9 (76)
AOR* (95% CI) [former vs never]				1.00 (Ref.)	0.99 (0.66–1.51)	1.98 (1.15–3.40)	2.63 (1.33–5.21)
AOR* (95% CI) [current vs never]				1.00 (Ref.)	1.42 (0.93–2.18)	3.31 (1.92–5.70)	4.12 (2.09–8.13)

IQR: interquartile range. AOR: adjusted odds ratio. Ref.: reference.

* A separate regression model was used to assess the association between the respective e-cigarette user characteristics (exposure variable) and dependence level (outcome variable), while adjusting for sex, nationality, and governorate of residence.

## DISCUSSION

The current study estimated the prevalence of e-cigarette use, assessed perceptions of e-cigarette harm and addictiveness, and measured dependence on e-cigarettes among adults living in Kuwait. This investigation estimated that 29.4% of the enrolled adults were current e-cigarette users and 19.1% were current cigarette smokers, with 14.0% being dual current users of e-cigarettes and cigarettes. A large proportion of participants reported that cigarettes (76.3%) and e-cigarettes (50.1%) are associated with ‘much harm’. Moreover, our data support the notion that e-cigarettes are perceived as ‘less harmful’ than cigarettes, as 40.6% of our sample reported such a perception. In reference to addictiveness, 41.8% of participants reported that e-cigarettes, relative to cigarettes, are ‘equally addictive’. Among current e-cigarette users, 84.8% were identified to have developed low, medium, or high dependence on e-cigarettes. These observations indicate that e-cigarettes are a substantial public health burden among adults in Kuwait, and preventive strategies are needed.

The prevalence of ever and current e-cigarette use in our study sample was estimated to be 40.2% and 29.4 %, respectively. Compared to other countries, in 2018, the prevalence of current e-cigarette use among US adults was estimated to be 5.5%^5^. In 2016, the prevalence of current e-cigarette use was estimated to be 4.8% among adults in Hong Kong^16^ and 3.2% among Malaysian adults^17^. In 2017, the prevalence of current e-cigarette use among individuals aged ≥15 years in 28 European Union countries was estimated to be 1.8%^18^. Regionally, the prevalence of e-cigarette use among college students in Saudi Arabia was estimated to be 27.7% in 2017^19^. A study among Saudi adults in 2019 estimated the prevalence of e-cigarette use to be 26.3%^20^. In Qatar, the current use of e-cigarettes was estimated to be 11.3% among adults in 2019^21^. In the United Arab Emirates, current e-cigarette use among young adults was estimated to be 23% in 2021^22^. Our findings indicate that the use of e-cigarettes among the enrolled adults in our study sample is elevated when compared to regional and international prevalence estimates. Such a disparity can be explained, at least partially, by the highly outreaching marketing campaigns that promoted e-cigarettes as a healthier alternative to tobacco smoking and as a smoking cessation product^23^. Moreover, their availability in a wide range of flavors has further increased their popularity. The lack of regulations and policies pertaining to e-cigarette marketing in Kuwait could explain the surge in e-cigarette use. Hence, public health policies and awareness campaigns are needed to curb such a surge in e-cigarette use.

Consistent with previous research^11^, a large proportion of the participants in our study perceived e-cigarettes as ‘less harmful’ than conventional cigarettes (40.6%). In contrast, 50.1% reported that e-cigarettes are associated with ‘much harm’. Our study showed that participants who never used e-cigarettes and cigarettes compared to current dual users were more likely to associate the use of e-cigarettes with ‘much harm’. Moreover, dual users compared to never users were more likely to perceive that e-cigarettes relative to cigarettes to be ‘less harmful’. This finding is consistent with a previous study that showed current smokers compared to former and never smokers are more likely to consider e-cigarettes as ‘less harmful’ than cigarettes^24^. In our study, more females than males reported that e-cigarettes are associated with ‘much harm’. Moreover, more males than females indicated that e-cigarettes relative to cigarettes are ‘less harmful’, which agrees with the findings of a previous study that found males to have greater odds than females to perceive e-cigarettes as ‘less harmful’^25^. Our study found that compared to cigarettes, 41.8% perceived that e-cigarettes are ‘equally addictive’, and 28.4% perceived them as ‘less addictive’. This finding is similar to a study among young US adults, which showed that 26.3% of their study participants agreed that e-cigarettes are less addictive than cigarettes^26^. Generally, such findings indicate that adults worldwide have similar perceptions of the harm and addictiveness of e-cigarettes relative to conventional cigarettes. This observation might reflect the unified marketing approach used by the e-cigarette industry globally. Hence, unified public health policies and regulations are needed to better inform the public about e-cigarettes.

Among current e-cigarette users in our study population, the most common reason for using e-cigarettes was ‘to quit using other tobacco products, such as cigarettes’, which is in agreement with the scientific literature^27^. This finding supports the notion that e-cigarettes were marketed as cessation products; however, their effectiveness as cigarette smoking cessation products is inconclusive^28,29^. Moreover, the majority of current e-cigarette users in our study sample reported using fruit flavor, which has also been the most commonly used flavor in other study samples^30^.

Dependence on e-cigarettes was measured among current users by using a previously developed standardized scale, namely the 10-item Penn State E-cigarette Dependence Index^14^. Among current e-cigarette users, the majority were classified as having developed ‘low dependence’ (41.3%), followed by ‘medium dependence’ (26.9%) and ‘high dependence’ (16.6%). Our analysis identified several factors that were associated with e-cigarette dependence levels. For instance, we found that using e-cigarettes for ≥1 year compared to <1 year was associated with medium and high dependence levels. Moreover, daily use of e-cigarettes compared to non-daily use of e-cigarettes was associated with low, medium, and high dependence levels. Also, the number of attempts to stop using e-cigarettes was found to be related to dependence levels, as such subjects who had fewer attempts to stop using e-cigarettes in the past 12-months compared to those who had frequent attempts to stop e-cigarettes use were more likely to have developed some level of dependence. These findings are in agreement with a prior study that showed daily use versus non-daily use and attempts to stop e-cigarette use to be associated with dependence levels^31^. Our results also showed that among current e-cigarette users, former cigarette smokers and current cigarette smokers (i.e. dual users), compared to those who never smoked cigarettes, had higher dependence levels. Previous studies have shown that the dependence on e-cigarettes varies according to cigarette smoking status^31,32^.

An important observation in our study was that the type of e-cigarette is associated with the dependence level, with users of pod-based e-cigarettes having the highest level of dependence and users of disposable e-cigarettes having the lowest level of dependence. A prior study found that individuals using pod-based e-cigarettes showed more frequent daily use and higher levels of e-cigarette dependence^33^. In another study, authors reported that pod-users versus non-pod users were more frequent users of e-cigarettes and had more signs of nicotine dependence^34^. Moreover, it has been shown that JUUL (a pod-based device) users compared to non-JUUL users, had greater odds of dependence and more frequent use^35^. These findings further support our observed increased dependence among current users of podbased e-cigarettes. It has been suggested that nicotine is not the only factor playing a role in addiction; as other additives that are administered simultaneously with nicotine have been shown to contribute to an increased risk of dependence such as smell, taste, and pleasant sensory stimuli which all play a role in increased dependence^36^.

### Strengths and limitations

A major strength of our study is the large sample size, which allowed us to estimate e-cigarettes use prevalence, perceptions of harm and addictiveness, and dependence levels among adults in Kuwait. Nonetheless, the applied snowball sampling technique, a non-random sampling method, may have yielded a study sample that is not representative of the total adults living in Kuwait, and hence the generalizability of our findings might not be applicable to the entire adult population in Kuwait. Moreover, selection bias cannot be eliminated due to the fact that participants needed access to a smartphone, tablet, or computer to be able to participate and complete the study questionnaire. Such selection bias can be seen in terms of the age of participants in our study, where the majority were aged 18–24 years (52.8%) and 25–34 (25.8%). Hence, interpretations of our findings should be made cautiously and may be reflective of young adults in Kuwait and not the general adult population. Nonetheless, in terms of education level, our study sample was similar to another nationally representative sample, with 59.5% of individuals in our study sample reported having a Bachelor’s degree or higher, and 60.2% of participants in a nationally representative sample reported having a Bachelor’s degree or higher^9^. Hence, such an observation indicates that our study sample is representative of the target adult population in Kuwait in terms of education level, which is a main determinant for smoking. A further limitation is that self-report of e-cigarette use might introduce information bias (measurement error). Adapting questions from the NYTS^12^ and the GATS^13^ standardized questionnaires facilitated comparisons with prior studies. Moreover, using the standardized 10-item Penn State E-cigarette Dependence Index is an added strength to our study, as it has been shown in a prior study that the dependence score calculated by the scale correlates strongly with nicotine concentration in e-liquids^14^. Dependence levels measured by the Penn State E-cigarette Dependence Index have also shown to be associated with indices that are indicative of dependence, such as self-perceptions of addiction to e-cigarettes, frequency of e-cigarette use, and time to first e-cigarette use in the morning^15^. Also, it is essential to note that all reported associations are cross-sectional (concurrent) hence no causal associations can be assumed, and the effect of selection bias and residual confounding cannot be eliminated.

## CONCLUSIONS

As the effect of e-cigarette use continues to be a public health concern, this study contributes important data and knowledge on e-cigarette use and perceptions among adults for the first time in Kuwait. This study showed that a large proportion of our study participants reported ever and current use of e-cigarettes. These findings indicate that patterns of e-cigarette use among adults in Kuwait follow, if not exceed, international trends, in which e-cigarettes are increasing in popularity, especially among youth and young adults. In addition, the majority of the participants perceived e-cigarettes as less harmful than conventional cigarettes. A substantial proportion of our study participants who reported current use of e-cigarettes were identified to have developed some level of dependence on e-cigarettes. Therefore, the knowledge of adults, including youth, regarding the possible harmfulness and addictiveness of e-cigarettes must be addressed at the population level. Worldwide, trends in the use of e-cigarettes must be followed regularly to better inform public health policies.

## Data Availability

The data supporting this research are available from the authors on reasonable request.
