# Supplementary material for: Prevalence of use, perceptions of harm and addictiveness, and dependence of electronic cigarettes among adults in Kuwait: A cross-sectional study
Source: Tob Induc Dis. 2023 Jul 14;21:90. doi: 10.18332/tid/163300 (PMC10347961; doi:10.18332/tid/163300)
Supplement: Supplementary file 1 [file TID-21-90-s1.pdf]

Table S1. Penn State Nicotine Dependence Index: Item Responses for Using Electronic Cigarettes among Current E-Cigarette Users (n = 892), Kuwait, 2021

**Items**	**% (n)**
1. How many times per day do you usually use your electronic cigarette? (assume that one "time" consists of around 15 puffs or lasts around 10 minutes)	
0-4 times/day	25.6 (228)
5-9 times/day	21.4 (191)
10-14 times/day	18.6 (166)
15-19 times/day	9.8 (87)
20-29 times/day	7.3 (65)
30+ times/day	17.4 (155)
2. On days that you can use your electronic cigarette freely, how soon after you wake up do you first use your electronic cigarette?	
0-5 minutes	33.0 (294)
6-15 minutes	18.0 (161)
16-30 minutes	12.7 (113)
31-60 minutes	12.9 (115)
61-120 minutes	8.1 (72)
121+ minutes	15.3 (137)
3. Do you sometimes awaken at night to use your electronic cigarette?	
Yes	12.7 (113)
No	87.3 (779)
4. If yes, how many nights per week do you typically awaken to use your electronic cigarette?†	
0-1 nights	46.0 (52)
2-3 nights	38.1 (43)
4+ nights	15.9 (18)
5. Do you use an electronic cigarette now because it is really hard to quit?	
Yes	35.0 (312)
No	65.0 (580)
6. Do you ever have strong cravings to use an electronic cigarette?	
Yes	51.8 (462)
No	48.2 (430)
7. Over the past week, how strong have the urges to use an electronic cigarette been?	
None/slight	34.3 (306)
Moderate/strong	47.7 (425)
Very strong/extremely strong	18.0 (161)
8. Is it hard to keep from using an electronic cigarette in places where you are not supposed to?	
Yes	29.7 (265)
No.	70.3 (627)
When you haven't used an electronic cigarette for a while or when you tried to stop using…	
9. Did you feel more irritable because you couldn't use an electronic cigarette?	
Yes	27.0 (241)
No	73.0 (651)
10. Did you feel nervous, restless, or anxious because you couldn't use an electronic cigarette?	
Yes	32.3 (288)
No	67.7 (604)

† Only subjects who answered "yes" to item #3 were asked to answer item #4 (i.e., n = 113).

© 2023 Alshaibani M. et al.
